# Supplementary material for: Eating Problems in Advanced Dementia: Navigating Difficult Conversations
Source: MedEdPORTAL. 2020 Nov 17;16:11025. doi: 10.15766/mep_2374-8265.11025 (PMC7678029; doi:10.15766/mep_2374-8265.11025)
Supplement: Supplementary file 1 — Facilitator Guide.docxParticipant Completed Worksheet.docxParticipant Handout.docxVideo.mp4Learning Objectives and Case.docxParticipant Blank Worksheet.docxParticipant Survey.docx [file mep_2374-8265.11025-s001.zip › A. Facilitator Guide.docx]

**Facilitator Guide**

**Welcome participants and introduce facilitator (2 minutes)**

Participants write their names on a card (optional)

**Facilitator shares the following agenda (~1 minutes)**

- Reviewing learning objectives (Appendix E)
- Introducing a clinical case in a paper format (Appendix E)
- Watching a trigger video (Appendix D) and discussing each question raised by the case depicted in the video
- Summary points and debriefing as well as handing out the completed worksheet
- Surveying participants

**Facilitator states the ground rules (~2 minutes)**

1. Respect others’ rights to hold opinions and beliefs that differ from your own. Challenge or criticize the idea, not the person.

2. Listen carefully to what others are saying even when you disagree with what is being said. Comments that you make (asking for clarification, sharing critiques, expanding on a point, etc.) should reflect that you have paid attention to the speaker’s comments.

3. Be courteous. Don’t interrupt or engage in private conversations while others are speaking.

4. Support your statements. Use evidence and provide a rationale for your points.

5. Allow everyone the chance to talk. If you have much to say, try to hold back a bit. If you are hesitant to speak, look for opportunities to contribute to the discussion.

6. If you are offended by something or think someone else might be, speak up and don't leave it for someone else to have to respond to it.

**Facilitator reads the learning objectives (Appendix E) (~2 minutes)**

**Facilitator or one participant reads the paper case (Appendix E) (~2 minutes)**

**The first trigger question is posed at the end of the written case: What would you do next?**

**Facilitator hands out the Participant blank worksheet (Appendix F) and allows 2 minutes after each of the following 9 trigger questions for participants to think about possible answers and write them down on the worksheet.**

**Facilitator promotes group discussion (~2 minutes)**

Understanding the natural history and the context of the development of the swallowing difficulty is the first step in the work-up. The provider needs more information that can be obtained in a family meeting.

**<< Start Video now >>**

**The video should be downloaded to the computer prior to starting the seminar and ready to play** to avoid delays caused by quality of internet connection. It takes about 5 minutes, depending on the internet connection speed, to download the video. The video can be played with any mp4 player (Quick Time Player for Mac, Media Player for Windows)**.**

**The rest of the trigger questions are embedded in the video.**

**Trigger question: Surrogate decision making: substituted judgment vs. best interest**

**<< Stop Video at 3:44 minutes >>**

**Facilitator stops video just after this trigger question appears in the video; allows 2 minutes for thinking /filling out the worksheet and promotes group discussion**

**Consider discussing: (~10 minutes)**

**Advance directives generally should take priority** over substituted judgment and best interest (in this order). Advance directives delineate who should act on the patients’ behalf and what should be done when the patient cannot make his/her own decisions.

Types of advance directives:

- Oral statements to family. Limitations -may be too general or not meant to be applied to the person. Not accepted by courts in few states.
- Oral statements to physicians- this is a better circumstance, particularly if recorded in the medical chart.
- Written directives:

1. Living wills delineate care given in case of a terminal condition. Large variations between states exist (for example, some states do not allow declining artificial hydration/nutrition through a living will).
2. Health care proxy- best way to assure one’s wishes are respected (along with having a statement about what life sustaining/other treatments are desired)
3. POLST-Physicians Orders for Life Sustaining Treatment- this form is filled out by the physician and patient based on end of life decisions.^1^ Once signed, doctors, emergency medical professionals, and other health care professionals must honor the instructions on this POLST form, no matter of place of care (at a hospital, care facility, home, etc.). In an emergency situation, any procedures that are legally required of emergency personnel will be overridden by personal decisions indicated on ones POLST. Other names depending of states may be: MOST (Medical Orders for Scope of Treatment), MOLST (Medical Orders for Life Sustaining Treatment), POST (Physician Orders for Scope of Treatment), TPOPP (Transportable Physician Orders for Patient Preferences). What is included: CPR, medical interventions (degree of care from full treatment to limited intervention to comfort care only), directive about hospitalization, medications such as antibiotics or medications for comfort and artificial hydration and nutrition. Major differences between a living will and a POLST: 1. POLST forms can instruct medical professionals about the care and treatments desired. Any emergency medical personnel and non-emergency medical professionals are legally obligated to follow the instructions of the POLST; they are not required to follow the instructions of a Living Will. 2. POLST forms address current, specific medical situations because POLST forms are only filled out when someone received a life limiting or serious diagnosis 3. POLST forms are “portable” between places of care because they are part of the medical record. Depending on the state of practice these forms may or may not be available – know your own states’ regulations.

Limitation to advance directives:

- Advance directives might not be informed (misconceptions about ventilators, CPR, overestimation of prognosis and reaction to treatments). People may not be able to develop preferences for various situations they have never been in.
- Patients may change their mind over time
- Problems interpreting advance directives
  - Vague terms such as “heroic measures”: what does that mean?
  - Applicability to similar situations (example: patient commented on stroke but now has dementia. How much leeway the surrogate has?)
  - Unrealistic situations
- Advance directives may conflict with pt.’s best interest (may wish to override if intervention short and good results, promising new therapies emerge or the person changed a lot over time)

**Rationale for discussing advance directives**: even very ill patients want to have these discussions with their physicians and want the physician to initiate the conversation. The Patient Self Determination Act of 1990, a federal mandate, was created to regulate the need to inform patients about their rights to provide advance directives; patients are not required to complete them.

A more recent initiative from the Institute for Healthcare Improvement and the John A. Hartford Foundation, meant to provide the best care to older adults and their caregivers, reduce harm and optimize the value of care for healthcare systems, is promoting particular attention to the following: carefully eliciting what matters to older adults (What Matters), are they on appropriate medications (Medications), what is the status of their cognition (Mentation) and mobility (Mobility). These comprise the 4 M’s of geriatrics.^2^

**Problems with discussing advance directives**: physicians usually discuss extreme scenarios but seldom the situations when prognosis is uncertain or disability is the outcome. Vague language, not eliciting knowledge about CPR/ventilator use, not using percentages/numbers for outcomes and not talking about other outcomes than full recovery or death are frequently cited problems.

**Improving discussions about advance directives:**

When should the discussion happen?

- When the course is uncertain, early in the course of disease and the physician/patient relationship.
- When there is a change in clinical status/new diagnosis.

Who should serve as decision maker?

What are the goals and values?

What are the preferences in specific situations?

- Physicians need to discuss likely scenarios and not extreme ones.
- Correct unrealistic expectations.

How should advance directives be interpreted? Elicit meaning of terms “heroic” for instance and ask about how much leeway the proxy/surrogate is given to interpret the situation.

How do patients want to be treated near the end of life?

**Continue advance directives discussions over time:**

Recommend written directives such as POLST if available

Document discussions in the medical record

Even when no advance directives are available, we still talk to the family most of the time. Issues arise when there is lack of consensus among family members or when placement is needed.

**Substituted judgment** needs to be applied when the patient gave no specific guidance but there is a decision maker.

**Problems with substituted judgment**:

-Inconsistency among proxy and other family members for example

-Inaccuracy (proxies will say what *they* want)

-Questionable considerations such as finances

-Unavoidable speculations

-Conflicts with patient’s best interest

**Best interest is applied** when no advance directives exist and substituted judgment is just a speculation. Ethically speaking the principle of beneficence obligates the physician to weight benefits and burdens of a procedure/treatment with the surrogate decision maker and act in the patients’ best interest.

Best interest may differ from previously expressed in the advance directives.

This does not mean the advance directives need to be ignored but the possibility of the person changing since the advance directives were written needs to be addressed. Did the person change so much that following the advance directives is not on his/her best interest? Was the circumstance of patient clinical status changing discussed with the surrogate? The advance directives should be followed unless there is a strong reason to override it.

**Problems with best interest**:

When surrogates make decisions based on own values or on financial aspects. However, surrogates cannot be expected to ignore their own needs/abilities completely.

Difficulties with faith-based requests (such as life should be prolonged even when suffering, to purify the person). The ethical guideline of non-maleficence allows providers to withhold procedures that may cause pain.^3^

**Facilitator starts the video when the above points discussed.**

**<< Restart Video at 3:44 minutes>>**

**Next trigger question appears in video:**

**Trigger question: How would you characterize the stage of Mr. Johnson’s dementia?**

**<< Stop Video at 5:28 minutes >>**

**Facilitator stops video at this point, allows 2 minutes for thinking /filling out the worksheet and promotes group discussion (~7 minutes)**

Dementia is diagnosed clinically based on the following (DSM V)

- Memory impairment
- One or more of the following (apraxia, aphasia, executive deficits)
- Functional impairment
- Deficits are present outside of delirium
- Deficits are not explained by a psychiatric condition

Using the description by SL Mitchell ^4^, a patient with advanced dementia is:

- - Dependent in all activities of daily living (ADLs)
  - Immobile
  - Unable to meaningfully communicate or recognize family members
  - Experience a high rate of infections due to general decline
  - Mini Mental State Examination (MMSE) score of ~5 or less, FAST Stage 7, Cognitive Performance Scale score of 6

Using the 7 stage FAST scale^5^

Stage 1 – normal adult

Stage 2 -very mild memory loss

Stage 3-early dementia/decreasing job and domestic function

Stage 4- mild dementia/Instrumental Activities of Daily Living (iADL) deficiency

Stage 5- moderate dementia/ADL deficiency

Stage 6- severe dementia/progression of ADL deficiency

6d- bladder incontinent

6e- bowel incontinent

*Stage 7- end stage dementia/ difficulty with ambulation, sitting, talking, smiling*

*7a- speech limited to few words*

*7b- speech limited to few words of less*

*7c- can’t walk w/o assistance*

*7d- can’t sit up w/o assistance*

*7e- unable to smile*

*7f- unable to hold head up independently*

Stage 7 or above is needed for hospice enrollment (among other criteria).

In the CASCADE study^4^ researchers followed prospectively 323 nursing home residents with advanced dementia in 22 nursing homes over 18 months. Key findings were:

- Dementia was under-recognized as a terminal illness
- Fifty-four percent of the residents died by the end of the follow up period; in the 3-month period preceding death, 90% of these residents developed an eating problem
- Dying trajectory in the last years of life was characterized by protracted functional impairment, profound aphasia, loss of mobility, incontinence and eating problems
- Eating problems were common (90 % of patients) and when eating problems developed the 6 months adjusted mortality rate was 39%.
- Aspiration events at the end of life were frequent – 41%.

**Facilitator starts the video when the above points discussed**

**<< Restart Video at 5:28 minutes>>**

**Next trigger question appears in video:**

**Trigger question: What other causes for eating difficulties you need to exclude?**

**<< Stop Video at 6:45 minutes>>**

**Facilitator stops video at this point, allows 2 minutes for thinking /filling out the worksheet and promotes group discussion (~5 minutes)**

In a study from 2009 by Vitale et al.^6^ the following were suggested for differential diagnosis of swallowing and eating problems:

- Delirium- with particular attention to the hypoactive delirium which may go unrecognized
- Poor oral hygiene/poor dentition
- Xerostomia (anticholinergics, mouth breathers, O2 use, Sjogren’s, dehydration)
- Esophageal dysmotility (neurological or GI conditions)
- Neuromotor dysfunction (CVA, progressive neurological disease, trauma
- Cervical osteophytes/ cervical spine surgery
- Zenker’s diverticulum
- COPD/CHF
- Head and neck cancer (pain/surgical changes)
- Loss of appetite (depression, infection, pain, nausea, delirium, constipation)
- Medication side effects

Be aware of swallowing changes that happen with age.^7^

- Age associated swallowing changes- presbyphagia increases with age and is a co-morbid condition in many chronic diseases and their treatments.
- Age related lingual pressure changes (the tongue is paramount in moving the bolus in the back of the throat) pressures are attained in elderly but slower.
- Age related oropharyngeal swallowing changes- “uncoupling “of voluntary oral swallowing from neurally controlled brainstem activity (closure of airway)

Swallowing co-morbidities:

- Xerostomia- older adults loose salivary producing acinar cells
- Esophageal motility-intra-esophageal stasis and reflux into upper structures (different than GERD)
- Sensory changes- decreased sensory discrimination in elderly. May explain why patients with dementia do not know what to do with bolus in the mouth. Texture and flavor enhancement may help.
- Sarcopenia- age related loss of muscle mass that may affect the muscles of the upper aero-digestive tract
- Medications (may affect swallowing via dry mouth, altering cognition, causing esophagitis)

**Medications producing xerostomia:**

Anticholinergics (sedating antihistamines, medications for Parkinson disease)

Antihypertensives (e.g. diuretics)

Opioids

Antipsychotics

**Medications altering cognition/alertness:**

Antianxiety

Antihypertensives (especially centrally acting)

Antiepileptics

Antiemetics

**Medications associated with esophagitis:**

Antibiotics

NSAID’s

Other (warfarin, diazepam, phenobarbital)

**Facilitator starts the video when the above points discussed.**

**<< Restart Video at 6:45 minutes >>**

**Next trigger question appears in video:**

**Trigger question: How would you respond here? What other tests are available?**

**<< Stop Video at 7:41 minutes >>**

**Facilitator stops video at this point, allows 2 minutes for thinking /filling out the worksheet and promotes group discussion (~5 minutes)**

- Bedside swallow evaluation by a speech pathologist provides information on:
- Postural and positioning characteristics
  - Oral motor function
  - Oral sensation
  - Vocal function
  - Response to food trials
  - Cognitive status

Caveat: the performance on this test does not always predict how the patient will be able to handle food presented to him/her.

- Modified barium swallow- video-fluoroscopic swallowing study indications
  - Evaluation of all stages of swallowing
  - Evaluation of swallowing physiology: base of tongue retraction; velopharyngeal closure; hyolaryngeal elevation; pharyngeal contraction; upper esophageal sphincter opening
  - **Measuring impact of therapeutic interventions on swallowing physiology**
  - Good study when upper esophageal dysfunction suspected
  - Good study when patient medically unfit or unwilling to participate in FEES
- Fiber endoscopic evaluation of swallowing (FEES) indications
  - High risk of aspiration
  - Evaluation of secretion management
  - Visualization of altered laryngopharyngeal anatomy/physiology
  - Impairment of laryngopharyngeal sensation is suspected
  - Extended examination to measure effects of fatigue or therapeutic interventions
  - **Evaluation with real food and fluid**
  - Biofeedback
  - Need for repeated swallowing examinations
  - Patient medically unfit or unwilling to participate in videofluoroscopy
  - **Patient unable/unsafe to sit**

FEES is a recognized tool for the assessment and management of swallowing disorders. It has been carried out by speech and language pathologists since its inception and description by Susan E. Langmore in 1988. It involves the trans-nasal insertion of a fiberoptic nasendoscope to the level of the oropharynx/hypopharynx to evaluate laryngopharyngeal physiology, management of secretions and the ability to swallow food and fluids. There is limited literature examining reliability of FEES. There are multiple factors that may affect the reliability of FEES interpretation including:

• The lack of validated and standardized rating scales and terminology

• Variable image quality due to equipment, experience of endoscopist and patient variables

• Lack of clinical information

• Level of experience of the assessing clinician

Deemed suitable in the elderly, should be avoided in patients with agitation.^8^

**Facilitator starts the video when the above points discussed.**

**<< Restart Video at 7:41 minutes >>**

**Next trigger question appears in video:**

**Trigger question: what is the evidence behind using a feeding tube in dementia?**

**<< Stop Video at 9:38 minutes >>**

**Facilitator stops video at this point, allows ~2 minutes for thinking /filling out the worksheet and promotes group discussion (7 minutes)**

**Feeding tube myths and potential benefits:**

- Feeding tubes improve survival in advanced dementia
- Feeding tubes prevent malnutrition/weight loss/starvation
- Feeding tubes help heal pressure ulcers
- Feeding tubes reduce incidence of aspiration pneumonia
- Feeding tubes prevent uncomfortable death

**Evidence behind survival in tube fed patients:**

- Long term survival benefits not shown in hospitalized patients receiving feeding tubes^9^
- Patients with advanced dementia in general have a high mortality with or without a feeding tube^4^
- Prospective cohort study of large sample of nursing home residents looked at one-year survival data (from MDS data set and Medicare data) after developing the need for feeding assistance; some of those residents received a feeding tube and some did not receive a feeding tube. The survival curves did not differ between the 2 groups. The timing of the feeding tube placement after developing the need for feeding assistance did not make any difference in the mortality. Median survival of this cohort was 177 days. Feeding tubes were found in 5 % on the cohort.^10^
- In a 2009 study by Teno and Kuo the mortality rate after feeding tube placement in patients with advanced dementia was 64%.^11^
- Additionally, if a naso-gastric tube was used for feeding, there was no difference in terms of survival.^12^
- A newer retrospective study^13^of 185 patients with a mean age of 76, a mixed cohort of probable Alzheimer dementia and schizophrenia showed that patients with tube feeding survived longer (695 days as opposed to 75 days if not tube fed) than those without tube feeding, even among dementia patients. The major criticism to this study showing longer survival with tube fed individuals is that patients in this study were categorized in earlier dementia stage (6e) and were hospitalized in a psychiatric unit for the duration of their lives where care might differ when compared to a home setting.

**Evidence behind malnutrition improvement in tube fed patients:**

In a review article from 2001^8^, a very small number of patients with feeding tubes had weight gain and improved nutritional parameters. Most of the studies showed no improvement in the markers. Even with adequate caloric intake some patients with dementia continued to lose weight.

- No good correlation in geriatric population between nutritional status and pressure ulcer healing.^14^
- Refeeding syndrome as a possible complication, with electrolyte imbalances, fluid retention.^12^
- A more recent review article concludes that there is data to support that feeding tubes help mitigate weight loss, sustain nutrition and reduce the suffering that occurs due to dehydration or malnutrition.^12^

**Evidence behind pressure ulcer healing in tube fed patients:**

- In this study by Teno, a propensity matched cohort of nursing home residents who were hospitalized, were monitored for pressure ulcer (stage 2 or higher) development. The cohort was carefully matched, 1 patient with a feeding tube to 3 without a feeding tube. The study found a 2 fold increased risk for development of stage II/ greater pressure ulcers in tube fed individuals with advanced dementia compared with those without a feeding tube and also lack of benefit in healing pressure ulcers.^10^ Feeding tubes may cause immobility via need for restraining the patient, hyperosmolar feedings can promote diarrhea, which can promote pressure ulcers. One caveat is that this study used administrative MDS data which has been found to be susceptible to over documentation bias.^12^

**Evidence behind aspiration pneumonia prevention in tube fed patients:**

- Having a feeding tube increases by 2-fold the development of aspiration pneumonia.^12^ In a cross-sectional study of nursing home residents, using MDS data, there was a strong association between the presence of a feeding tube and development of aspiration pneumonia.^15^
- The presence of a feeding tube increases the risk of gram-negative bacilli colonization of the mouth compared with hand fed individuals, which in turn increases the risk of pneumonia.^12^
- Other predictors of increased risk for aspiration pneumonia: frequent suctioning, COPD/CHF, bedfast status, nursing home high case mix index, delirium, weight loss, swallowing problems, UTI’s, mechanically altered diet, dependence for eating, bed mobility, locomotion, number of medications, age.
- Some data is supporting reduced incidence of aspiration pneumonia and prolonged survival for more than 2 years on older Japanese patients with dementia (when compared with a naso-gastric tube fed individuals.^12^

**Evidence behind increased comfort in tube fed patients:**

- Malfunctioning tubes and need for re-insertion are common (20%) in older adults with advanced dementia resulting frequent ER visits.^11^
- Nursing home residents with a feeding tube and advanced dementia spend more time in the ICU, then their counterparts without a feeding tube.^16^
- In a follow up study of the CASCADE by SL Mitchell, up **47% of ER** visits were caused by feeding tube related complications.^17^

**Evidence behind functional improvement in tube fed patients**: no data to suggest results.^14^

- Carey et al in 2006 looked at proxy expectations vs. outcomes of patients who received a feeding tube (CVA, ENT, 16 % of this cohort had a neurodegenerative disease, other serious illnesses). At baseline this was an impaired group with a mean ADL score of 11(5-15). Impairment in most ADL’s continued at 6 months after a feeding tube was placed. 1 in 5 patients died at 3 months. Six months’ mortality rate was 30 % observed.^18^

**Cost burden of feeding tubes:**

- Feeding tube is costlier than hand feeding (total cost billed to Medicare in tube fed patient sis $6994 versus $959 for hand fed patients).^12^

**Other potential benefits in tube fed patients:**

- Easing caregiver burden due to tension, anxiety around mealtimes and guilt about starvation when the patient with dementia is consistently refusing to take in nutrition.^12^

As part of the ABIM Foundation Choosing Wisely campaign meant to promote patient-physician conversations, the American Geriatrics Society developed a list of 10 items to consider when discussing the care of older adults^19^. Amongst those there is a recommendation to avoid feeding tube insertion in patients with advanced dementia but rather choose careful hand feeding instead. You can reiterate this statement for the last trigger question discussion.

The decision for feeding tube placement in advanced dementia should be made on case by case basis and should take in consideration the evidence supporting burdens but also its potential benefits.

**Facilitator starts the video when the above points discussed.**

**<< Restart Video at 9:38 minutes >>**

**Next trigger question appears in video:**

**Trigger question: What are the common complications of feeding tube placement?**

**<< Stop Video at 10:14 minutes >>**

**Facilitator stops video at this point, allows 2 minutes for thinking /filling out the worksheet and promotes group discussion (~3-5 minutes)**

**Complications and risks of feeding tube placement:**

- Low procedure related mortality rate **1%-2% (**arrhythmia, bleeding, perforation into abdominal cavity)
- Possible complications: wound infection, aspiration pneumonia, sinus and middle ear infections, bleeding, leakage, tube occlusion, erosion into abdominal wall, abdominal wall abscess, necrotizing fasciitis, colo-cutaneous fistula, ileus, diarrhea, nausea, vomiting, increased GERD, aspiration, metabolic and electrolyte imbalances with re-feeding, restraint use, loss of social interaction with feeding.
- Mortality after feeding tube placement:
  - Thirty-day mortality **28%** and **6**-month mortality is **52%** in a study by Sanders^20, 12, 21^
- Other deleterious effects
- Increased rates of care transitions such as hospital transfers and ED visits^14^

**Facilitator starts the video when the above points discussed.**

**<< Restart Video at 10:14 minutes >>**

**Next trigger question appears in video:**

**Trigger question: What is the value of supplements and appetite stimulants in managing eating problems in patients with advanced dementia?**

**<< Stop Video at 10:56 minutes >>**

**Facilitator stops video at this point, allows ~2 minutes for thinking /filling out the worksheet and promotes group discussion (~3-5 minutes)**

A systematic literature search identified randomized trials with low to medium risk of bias about oral feeding options in people with dementia. (Limitations: heterogeneous population in regards to dementia stage and feeding problems, interventions and outcomes).

Looked at:

- High calorie supplements: moderate evidence to support improvement in weight, BMI but low evidence for improved wound healing, reducing risk of infections.
- Assisted feeding options: low evidence that improves weight.
- Other initiatives: appetite stimulants (Megace, Dronabinol), modified food consistency: low evidence for weight improvement.

No effect of any of the above on function, cognition and mortality in people with moderate to severe dementia.^22^

Optimal timing of nutritional interventions remains unclear: may be ineffective when initiated before nutrition becomes a major problem or in advanced dementia cases and low BMI when it may be too late.

It is difficult to study this topic due to ethical concerns about withholding feeding treatments.

Other intervention strategies are suggested:

1. Compensatory measures such as:

• Postural adjustments

• Food and liquid rate and amount

• Diet modification

• Adaptive equipment

1. Rehabilitative- active exercises
2. Occasionally surgical procedures such as cricopharyngeal myotomy, Zenker’s diverticula removal
3. Oral hygiene- several times per day, relieve oral dryness

**Facilitator starts the video when the above points discussed.**

**<< Restart Video at 10:56 minutes >>**

**Next trigger question appears in video:**

**Trigger questions: Is he going to starve to death?**

**<< Stop Video at 12:47 minutes >>**

**Facilitator stops video at this point, allows 2 minutes for thinking / filling out the worksheet and promotes group discussion (3-5 minutes)**

A study of 32 mentally awake, competent patients with a terminal illness for symptom of hunger, thirst and dry mouth showed that most of them (63%) never experienced hunger. Thirst was more common initially (62%). All the above could be alleviated with small amounts of food fluid or ice chips/lubrication to the lips.^23^

How to better approach the question of “feeding tube or no feeding tube”?

- Emphasize evidence base for feeding tubes including burdens and potential benefits.
- Use decision aides. This study by Snyder showed improved knowledge scores in caregivers, decreased expectations and reduced decisional conflict^24^
- A more recent study by Mitchell, focused on burdensome care in advanced dementia, showed that an advance care planning video did impact surrogate decision-making regarding tube feeding.^25^
- A Decision Aide for caregivers: Improving Decision Making About Feeding Options in Dementia on Vimeo.^26^ This is a 19-minute video available for free on Vimeo for surrogate decision makers.
- Reframing the conversation: Introduce the concept of comfort feeding - focused on quality of life, value of feeding as nurturing, the natural way of providing nutrition to the body.^27^

**Facilitator starts the video when above points discussed.**

**<< Restart Video at 12:47 minutes >>**

**This concludes the video, which is 14:49 minutes long.**

**Facilitator should consider the following debriefing questions**: (~3-5 minutes)

Regarding aspects of the provider-family interaction:

What went well? What did not go so well?

How was the non-verbal communication?

Did this encounter trigger emotion in you? Would you be willing to talk about them?

What other strategies would you have used?

What have you seen others do and may have worked well here?

**Facilitator hands out participant survey (Appendix G) and collects completed surveys (~5 minutes) and hands out the Participant handout (Appendix C)**

**This concludes the workshop.**

**References**

1. Home-POLST <https://polst.org/>
2. Age-Friendly Health Systems <https://www.johnahartford.org/age-friendly-health-systems-initiative>
3. Smith AK, Lo B, Sudore R. When previously expressed wishes conflict with best interests. JAMA Intern Med. 2013;173(13):1241-1245. doi:10.1001/jamainternmed.2013.6053
4. Mitchell SL, Teno JM, Kiely DK, et al. The clinical course of advanced dementia. N Engl J Med. 2009;361(16):1529-1538. doi:10.1056/NEJMoa0902234
5. Reisberg B. Functional assessment staging (FAST). Psychopharmacol Bull. 1988;24(4):653-659.
6. Vitale CA, Monteleoni C, Burke L, Frazier-Rios D, Volicer L. Strategies for improving care for patients with advanced dementia and eating problems. Annals of Long Term Care.2009;17(5):32-39.
7. Ney DM, Weiss JM, Kind AJ, Robbins J. Senescent swallowing: impact, strategies, and interventions. Nutr Clin Pract. 2009;24(3):395-413. doi:10.1177/0884533609332005
8. Fibreoptic Endoscopic Evaluation of Swallowing (FEES): The role of speech and language therapy. https://www.dysphagie-netzwerk-suedwest.de/wp-content/uploads/2016/09/FEES_Standard_Rosenbek1996.pdf
9. Meier DE, Ahronheim JC, Morris J, Baskin-Lyons S, Morrison RS. High short-term mortality in hospitalized patients with advanced dementia: lack of benefit of tube feeding. Arch Intern Med. 2001;161(4):594-599. doi:10.1001/archinte.161.4.594
10. Teno JM, Gozalo P, Mitchell SL, Kuo S, Fulton AT, Mor V. Feeding tubes and the prevention or healing of pressure ulcers. Arch Intern Med. 2012;172(9):697-701. doi:10.1001/archinternmed.2012.1200
11. Kuo S, Rhodes RL, Mitchell SL, Mor V, Teno JM. Natural history of feeding-tube use in nursing home residents with advanced dementia. J Am Med Dir Assoc. 2009;10(4):264-270. doi:10.1016/j.jamda.2008.10.010
12. Ijaopo EO, Ijaopo RO. Tube Feeding in Individuals with Advanced Dementia: A Review of Its Burdens and Perceived Benefits. J Aging Res. 2019;2019:7272067. Published 2019 Dec 19. doi:10.1155/2019/7272067
13. Takayama K, Hirayama K, Hirao A, et al. Survival times with and without tube feeding in patients with dementia or psychiatric diseases in Japan. Psychogeriatrics. 2017;17(6):453-459. doi:10.1111/psyg.12274
14. Finucane TE, Christmas C, Travis K. Tube feeding in patients with advanced dementia: a review of the evidence. JAMA. 1999;282(14):1365-1370. doi:10.1001/jama.282.14.1365
15. Langmore SE, Skarupski KA, Park PS, Fries BE. Predictors of aspiration pneumonia in nursing home residents. Dysphagia. 2002;17(4):298-307. doi:10.1007/s00455-002-0072-5
16. Teno JM, Gozalo PL, Bynum JP, et al. Change in end-of-life care for Medicare beneficiaries: site of death, place of care, and health care transitions in 2000, 2005, and 2009. JAMA. 2013;309(5):470-477. doi:10.1001/jama.2012.207624
17. Givens JL, Selby K, Goldfeld KS, Mitchell SL. Hospital transfers of nursing home residents with advanced dementia. J Am Geriatr Soc. 2012;60(5):905-909. doi:10.1111/j.1532-5415.2012.03919.x
18. Carey TS, Hanson L, Garrett JM, et al. Expectations and outcomes of gastric feeding tubes. Am J Med. 2006;119(6):527.e11-527.e5.27E16. doi:10.1016/j.amjmed.2005.11.021
19. Choosingwisely.org https://www.choosingwisely.org/wp-content/uploads/2018/02/Feeding-Tubes-For-People-With-Alzheimers-AGS.pdf
20. Sanders DS, Carter MJ, D'Silva J, James G, Bolton RP, Bardhan KD. Survival analysis in percutaneous endoscopic gastrostomy feeding: a worse outcome in patients with dementia. Am J Gastroenterol. 2000;95(6):1472-1475. doi:10.1111/j.1572-0241.2000.02079.x
21. Goldberg LS, Altman KW. The role of gastrostomy tube placement in advanced dementia with dysphagia: a critical review. Clin Interv Aging. 2014;9:1733-1739. Published 2014 Oct 14. doi:10.2147/CIA.S53153
22. Hanson LC, Ersek M, Gilliam R, Carey TS. Oral feeding options for people with dementia: a systematic review. J Am Geriatr Soc. 2011;59(3):463-472. doi:10.1111/j.1532-5415.2011.03320.x
23. McCann RM, Hall WJ, Groth-Juncker A. Comfort care for terminally ill patients. The appropriate use of nutrition and hydration. JAMA. 1994;272(16):1263-1266. doi:10.1001/jama.272.16.1263
24. Snyder EA, Caprio AJ, Wessell K, Lin FC, Hanson LC. Impact of a decision aid on surrogate decision-makers' perceptions of feeding options for patients with dementia. J Am Med Dir Assoc. 2013;14(2):114-118. doi:10.1016/j.jamda.2012.10.011.
25. Mitchell SL, Shaffer ML, Cohen S, Hanson LC, Habtemariam D, Volandes AE. An Advance Care Planning Video Decision Support Tool for Nursing Home Residents With Advanced Dementia: A Cluster Randomized Clinical Trial. JAMA Intern Med. 2018;178(7):961-969. doi:10.1001/jamainternmed.2018.1506
26. Improving Decision-Making about Feeding Options in Dementia <https://vimeo.com/51776155>
27. Palecek EJ, Teno JM, Casarett DJ, Hanson LC, Rhodes RL, Mitchell SL. Comfort feeding only: a proposal to bring clarity to decision-making regarding difficulty with eating for persons with advanced dementia. J Am Geriatr Soc. 2010;58(3):580-584. doi:10.1111/j.1532-5415.2010.02740.x
