# Supplementary material for: Eating Problems in Advanced Dementia: Navigating Difficult Conversations
Source: MedEdPORTAL. 2020 Nov 17;16:11025. doi: 10.15766/mep_2374-8265.11025 (PMC7678029; doi:10.15766/mep_2374-8265.11025)
Supplement: Supplementary file 1 — Facilitator Guide.docxParticipant Completed Worksheet.docxParticipant Handout.docxVideo.mp4Learning Objectives and Case.docxParticipant Blank Worksheet.docxParticipant Survey.docx [file mep_2374-8265.11025-s001.zip › B. Participant Completed Worksheet.docx]

| **When Eating problems Arise in Advanced Dementia: Navigating Difficult Conversations with Caregivers** | | | | |
| --- | --- | --- | --- | --- |
| **Trigger question** | **What is the provider worried about?** | **What is the family worried about?** | **What do we say?** | **What do we do?** |
| Surrogate decision makers (SDM): Substituted Judgment vs. Patients’ Best Interest | Providers are concerned about existence of an advance directive/ decision maker(s) and also if SDM’s are following patient wishes, if expressed in the past. | We have never talked about his/her wishes! | Care should be provided in accordance with patient expressed wishes, if known.  If wishes not known we can help you figure out what would he/she want, keeping in mind his/her best interest. | Physicians should actively participate in decision-making, based on prognostic data and prior clinical experience. ^1^ |
| How would you characterize the patients’ stage of dementia? | Is this a patient in the advanced stages of dementia?  Is the dementia causing progressive oro-pharyngeal dysphagia? | No one told us before about the relationship between eating and dementia. | We’d like to better understand the course of the patients’ cognitive problems as well as explore observations made by caregivers and healthcare providers during eating | Explore history of cognitive loss. Understand the nature/course of the eating problem. Educate family about what to expect in advanced dementia. ^2^ |
| What other causes for eating difficulty you need to exclude? | Any reversible causes of poor oral intake contributing? | What can be done to improve eating? | We will look carefully for any other contributing factors than can make this problem worse. | Assess for delirium and any oral dental, laryngeal or esophageal problem. Do a thorough medication review. Correct reversible causes. ^3^ |
| What other tests are available? | Be able to discuss with surrogate decision maker (SDM) what other tests exist and why those tests are not usually recommended  (modified barium swallow, endoscopic evaluation) | Will you do another test to confirm the problem?  (Are they giving up on my father?) | These tests can be difficult to perform and interpret and usually do not add to what can be obtained with a bedside swallowing evaluation. | All these tests depend on patient cooperation. |

| What is the evidence behind using a feeding tube in patients with dementia? | Feeding tubes are not indicated in patients with advanced dementia.  (How will I be able to convey this to his family?) | How is he/she going to eat? | Placement of a feeding tube will not impact the course of his/her dementia.  “Instead we recommend comfort feeding through careful hand feeding” | Discuss myths in relation to feeding tube placement (preventing aspiration, improving survival, healing pressure sores, improving function) ^4^ |
| --- | --- | --- | --- | --- |
| What are the common complications of feeding tube placement? | Providers worried about burden exceeding benefits | Is there any harm in trying? | We find that the level of overall discomfort that patients such as your father often experience related to a feeding tube can be substantial. | Discuss continued aspiration, insertion site discomfort, local infection, accidental removal, and agitation/restraint use, diarrhea, and development of a pressure ulcer. Increased transitions to ED/hospital.  If family insisting of feeding tube placement can discuss timed trial of either an NG tube or a feeding tube.  Bib: ^5^ |
| What is the value of supplements and appetite stimulants in managing eating problems in patients with advanced dementia? | Recent literature review shows limited benefit for supplements and appetite stimulants | Can you give him/her something to stimulate appetite? | Supplements and appetite stimulants showed little help with weight but no meaningful help with function. Did not prolong survival. | Weight and BMI can be impacted but no data to support improvement in function, healing pressure ulcers, no improved survival. ^6^ |
| Will he/she starve to death? | Providers worry about needing to talk about death /dying. Nursing home providers worry about maintaining nutritional markers (weight, albumin) | Are we starving him/her to death? | No, he is not starving. Your loved one is getting to where he /she needs much less food to survive. We can provide food for comfort, plan for consequences of low albumin (med dose) | Can mention what we know from terminally ill patients needing less food and fluids. ^7^  Involve SLP for best food consistency, positioning, assistive devices, and pharmacist to help adjust medications.  Family to help with food preferences (ethnic foods) |

Authors: Erika Manu MD, Caroline Vitale MD, University of Michigan. Format adapted with permission from Dr. Kenneth Pituch MD, University of Michigan

**Bibliography:**

1. Smith AK, Lo B, Sudore R. When previously expressed wishes conflict with best interests. JAMA Intern Med. 2013 Jul 8; 173(13): 1241-5. PMID: 23712743.

2. Mitchell SL, Teno JM, Kiely DK, Shaffer ML, Jones RN, Prigerson HG, Volicer L, Givens JL, Hamel MB. The clinical course of advanced dementia. N Engl J Med. 2009 Oct 15; 361(16): 1529-38. PMID: 19828530.

3. Vitale CA, Monteleoni C, Burke L, Frazier-Rios D, Volicer L. Strategies for improving care for patients with advanced dementia and eating problems. Annals of Long Term Care.2009;17(5):32-39.

4. See bibliography 1-8 in Appendix C.

5. See bibliography 1-8 in Appendix C.

6. Hanson LC, Ersek M, Gilliam R, Carey TS. Oral feeding options for people with dementia: a systematic review. J Am Geriatr Soc. 2011 Mar; 59(3): 463-72. PMID: 21391936.

7. McCann RM, Hall WJ, Groth-Juncker A. Comfort care for terminally ill patients. The appropriate use of nutrition and hydration. JAMA. 1994 Oct 26; 272(16): 1263-6. PubMed PMID: 7523740.
