# Supplementary material for: Eating Problems in Advanced Dementia: Navigating Difficult Conversations
Source: MedEdPORTAL. 2020 Nov 17;16:11025. doi: 10.15766/mep_2374-8265.11025 (PMC7678029; doi:10.15766/mep_2374-8265.11025)
Supplement: Supplementary file 1 — Facilitator Guide.docxParticipant Completed Worksheet.docxParticipant Handout.docxVideo.mp4Learning Objectives and Case.docxParticipant Blank Worksheet.docxParticipant Survey.docx [file mep_2374-8265.11025-s001.zip › C. Participant Handout.docx]

**Approach to Feeding Decisions in Advanced Dementia**

Place eating and swallowing problems into appropriate context

(hallmark of end-stage dementia)

Ascertain primary goals of care by engaging surrogate / understanding patient preferences when known

Careful clinical assessment looking for reversible factors of eating and swallowing problems

Present eating/feeding options (with known risks and benefits)

Align options with goals of care

Provide ongoing decision support to family / caregivers

**Feeding tube placement myths and realities in advanced dementia**

*Does a feeding tube…*

Prevent aspiration?

No, having a feeding tube actually predicts development of aspiration pneumonia. ^1^

Improve pressure ulcer healing?

No, actually the presence of a feeding tube increases the risk of development of stage II and greater pressure ulcers.^2^

Prolong survival?

Nursing home residents who developed the need for feeding assistance (some did receive a feeding tube, some did not). The 1- year survival rate was the same between the groups.^3^

While some newer observational studies show improved survival^4^ multiple observational studies show 30- 60% mortality rate at 6 months after feeding tube placement.^3,6^

Improve functional status?

No, after a feeding tube was placed ADL’s remained impaired at 6 months follow up.^5^

Improve comfort?

Feeding tube related complications in patients with advanced dementia caused 47% of ER visits in this population.^6,7^ These visits have been found uncomfortable for this patient population.

Newer data suggest that feeding tubes help mitigate weight loss, sustain nutrition and reduce the suffering that occurs due to dehydration or malnutrition.^8^

**AGS Choosing Wisely^®^**

**An Inntiative of the ABIM Foundation**

**Ten things Clinicians and Patients Should Question** ^9^



Manu, Erika

AGS:Don’t recommend percutaneous feeding tubes in patients with advanced dementia; instead offer oral assisted feeding. Careful hand-feeding for patients with severe dementia is at least as good as tube-feeding for the outcomes of death, aspiration pneumonia,

4:47 PM

**Don’t recommend percutaneous feeding tubes in patients with advanced dementia; instead offer oral assisted feeding.**

Careful hand feeding for patients with severe dementia is at least as good as tube feeding for the outcomes of death, aspiration pneumonia, functional status and patient comfort. Food is the preferred nutrient. Tube feeding is associated with agitation, increased use of physical and chemical restraints and worsening pressure ulcers.

**Medications affecting swallowing** ^10^

**Medications producing xerostomia:**

Anticholinergics (sedating antihistamines, medications for Parkinson disease)

Antihypertensives (eg. diuretics)

Opioids

Antipsychotics

**Medications altering cognition/alertness:**

Antianxiety

Antihypertensives (especially centrally acting)

Antiepileptics

Antiemetics

**Medications associated with esophagitis:**

Antibiotics

NSAID’s

Other (warfarin, diazepam, phenobarbital)

**Bibliography:**

1.Langmore SE, Skarupski KA, Park PS, Fries BE. Predictors of aspiration pneumonia in nursing home residents. Dysphagia. 2002, 17(4): 298-307.

2. Teno JM, Gozalo P, Mitchell SL, Kuo S, Fulton AT, Mor V. Feeding tubes and the prevention or healing of pressure ulcers. Arch Intern Med. 2012 May14; 172(9): 697-701.

3. Teno JM, Gozalo PL, Mitchell SL, Kuo S, Rhodes RL, Bynum JP, Mor V. Does feeding tube insertion and its timing improve survival? J Am Geriatr Soc. 2012 Oct; 60(10): 1918-21.

4.Takayama, K., Hirayama, K., Hirao, A., Kondo, K., Hayashi, H., Kadota, K., Asaba, H., Ishizu, H., Nakata, K., Kurisu, K., Oshima, E., Yokota, O., Yamada, N., & Terada, S. (2017). Survival times with and without tube feeding in patients with dementia or psychiatric diseases in Japan. *Psychogeriatrics*, *17*(6), 453-459.

5. Carey TS, Hanson L, Garrett JM, Lewis C, Phifer N, Cox CE, Jackman A. Expectations and outcomes of gastric feeding tubes. Am J Med. 2006 Jun; 119(6): 527.e11-6.

6. Kuo S, Rhodes RL, Mitchell SL, Mor V, Teno JM. Natural history of feeding-tube use in nursing home residents with advanced dementia. J Am Med Dir Assoc. 2009 May; 10(4): 264-70.

7. Givens JL, Selby K, Goldfeld KS, Mitchell SL. Hospital transfers of nursing home residents with advanced dementia. J Am Geriatr Soc. 2012 May; 60(5):905-9.

8. Ijaopo EO, Ijaopo RO. Tube Feeding in Individuals with Advanced Dementia: A Review of Its Burdens and Perceived Benefits. J Aging Res. 2019;2019:7272067. Published 2019 Dec 19. doi:10.1155/2019/7272067

9 Choosingwisely.org https://www.choosingwisely.org/wp-content/uploads/2018/02/Feeding-Tubes-For-People-With-Alzheimers-AGS.pdf

10. Senescent Swallowing: Impact, Strategies and Interventions. Nutrition in clinical practice: official publication of the American Society for Parenteral and Enteral Nutrition. 2009; 24(3): 395-413
