# Supplementary material for: Eating Problems in Advanced Dementia: Navigating Difficult Conversations
Source: MedEdPORTAL. 2020 Nov 17;16:11025. doi: 10.15766/mep_2374-8265.11025 (PMC7678029; doi:10.15766/mep_2374-8265.11025)
Supplement: Supplementary file 1 — Facilitator Guide.docxParticipant Completed Worksheet.docxParticipant Handout.docxVideo.mp4Learning Objectives and Case.docxParticipant Blank Worksheet.docxParticipant Survey.docx [file mep_2374-8265.11025-s001.zip › E. Learning Objectives and Case.docx]

**When Eating Problems Arise in Patients with Dementia**

**Learning Objectives:**

1. Review importance of completing advance directives (applied to patients with dementia); define surrogate decision maker, substituted judgment, and best interest concepts and discuss advance care planning for the case presented.
2. Describe the natural history of advanced dementia.
3. Become familiar with potential treatment burdens associated with tube feeding in patients with advanced dementia.
4. Appreciate various options for eating/feeding in advanced dementia and swallowing problems.
5. Analyze aspects of difficult conversations with surrogate decision makers.

**The case**

Mr. Johnson is an 83-year-old male with a past medical history significant for hypertension, Alzheimer’s dementia, heart disease with coronary artery bypass surgery 15 years ago, dyslipidemia, hypothyroidism and osteoarthritis, who was admitted to your service last night from the emergency room. A note accompanied him from the nursing home stating that he has been eating less and less each day and that he now appears generally weak. His daughter, who accompanied him to the emergency room, explained to the physician that Mr. Johnson has been losing weight since he was last hospitalized few months ago with pneumonia. In the emergency room lab values revealed a hemoglobin of 11.3 mg/dl, white count of 10,000 with no neutrophilia, BUN 32 mg/dl, creatinine of 2.1 (baseline 1.2) mg/dl and albumin of 2.3 g/dl. Urinalysis was unremarkable except for the urine appearing concentrated. He was admitted for IV hydration. On hospital day # 2, his labs are trending in the right direction but his nurse pulls you aside and informs you that she witnessed him coughing and nearly choking on food while she was trying to assist him with his meal. She is worried that he may be aspirating and asks you to consider obtaining a swallowing evaluation.

**What would you do next?**
