# Supplementary material for: Eating Problems in Advanced Dementia: Navigating Difficult Conversations
Source: MedEdPORTAL. 2020 Nov 17;16:11025. doi: 10.15766/mep_2374-8265.11025 (PMC7678029; doi:10.15766/mep_2374-8265.11025)
Supplement: Supplementary file 1 — Facilitator Guide.docxParticipant Completed Worksheet.docxParticipant Handout.docxVideo.mp4Learning Objectives and Case.docxParticipant Blank Worksheet.docxParticipant Survey.docx [file mep_2374-8265.11025-s001.zip › F. Participant Blank Worksheet.docx]

| **When Eating problems Arise in Advanced Dementia: Navigating Difficult Conversations with Caregivers** | | | | |
| --- | --- | --- | --- | --- |
| **Trigger question** | **What is the provider worried about?** | **What is the family worried about?** | **What do we say?** | **What do we do?** |
| Surrogate decision makers (SDM): Substituted Judgment vs. Patients’ Best Interest |  |  |  |  |
| How would you characterize the patients’ stage of dementia? |  |  |  |  |
| What other causes for eating difficulty you need to exclude? |  |  |  |  |
| What other tests are available? |  |  |  |  |

| What is the evidence behind using a feeding tube in patients with dementia? |  |  |  |  |
| --- | --- | --- | --- | --- |
| What are the common complications of feeding tube placement? |  |  |  |  |
| What is the value of supplements and appetite stimulants in managing eating problems in patients with advanced dementia? |  |  |  |  |
| Will he/she starve to death? |  |  |  |  |

Authors: Erika Manu MD, Caroline Vitale MD, University of Michigan. Format adapted with permission from Dr. Kenneth Pituch MD, University of Michigan
