# Supplementary material for: Eating Problems in Advanced Dementia: Navigating Difficult Conversations
Source: MedEdPORTAL. 2020 Nov 17;16:11025. doi: 10.15766/mep_2374-8265.11025 (PMC7678029; doi:10.15766/mep_2374-8265.11025)
Supplement: Supplementary file 1 — Facilitator Guide.docxParticipant Completed Worksheet.docxParticipant Handout.docxVideo.mp4Learning Objectives and Case.docxParticipant Blank Worksheet.docxParticipant Survey.docx [file mep_2374-8265.11025-s001.zip › G. Participant Survey.docx]

**When Eating Problems Arise In Patients with Dementia: Navigating Difficult Conversations with Caregivers**

**Self-assessment of your skills:** For each statement below, please rate your skill level prior to and after participating in this workshop.

*(Please check the box that best describes your skill level both BEFORE and AFTER completing this course.)*

**BEFORE completing the workshop AFTER completing the workshop**

**Needed further With With Need further With With**

**I could… instruction supervision back-up Unsupervised instruction supervision back-up Unsupervised**

| 1. Conduct an appropriate investigation of the eating problem in patients with advanced dementia. | **⬜ 1** | **⬜ 2** | **⬜ 3** | **⬜ 4** | **⬜ 1** | **⬜ 2** | **⬜ 3** | **⬜ 4** |
| --- | --- | --- | --- | --- | --- | --- | --- | --- |
| 2. Discuss eating problems with surrogate decision makers of patients with dementia. | **⬜ 1** | **⬜ 2** | **⬜ 3** | **⬜ 4** | **⬜ 1** | **⬜ 2** | **⬜ 3** | **⬜ 4** |
| 3. Discuss treatment burdens associated with feeding tube placement in patients with advanced dementia. | **⬜ 1** | **⬜ 2** | **⬜ 3** | **⬜ 4** | **⬜ 1** | **⬜ 2** | **⬜ 3** | **⬜ 4** |
| 4. Discuss alternatives to feeding tube placement. | **⬜ 1** | **⬜ 2** | **⬜ 3** | **⬜ 4** | **⬜ 1** | **⬜ 2** | **⬜ 3** | **⬜ 4** |
| 5. Discuss ethical issues surrounding artificial feeding in patients with dementia. | **⬜ 1** | **⬜ 2** | **⬜ 3** | **⬜ 4** | **⬜ 1** | **⬜ 2** | **⬜ 3** | **⬜ 4** |

**In this section, we would like your assessment of the perceived educational impact of the workshop: *(****Please check the appropriate box.)*

| 7. Please rate the importance of the workshop topic. | | | |
| --- | --- | --- | --- |
| Not at all important  ⬜ 1 | Somewhat important  ⬜ 2 | Important  ⬜ 3 | Very important  ⬜ 4 |
| 8. Please rate the usefulness of the interactive aspects of the workshop. | | | |
| Not at all useful  ⬜ 1 | Somewhat useful  ⬜ 2 | Useful  ⬜ 3 | Very useful  ⬜ 4 |
| 9. Please rate the educational value of the workshop. | | | |
| No value  ⬜ 1 | Some value  ⬜ 2 | Valuable  ⬜ 3 | Very valuable  ⬜ 4 |
| 10. Please rate your willingness to recommend this workshop to other trainees. | | | |
| Not recommend  ⬜ 1 | May recommend  ⬜ 2 | Recommend  ⬜ 3 | Highly recommend  ⬜ 4 |
| 11. Please rate the effectiveness of the moderator(s). | | | |
| Not effective  ⬜ 1 | Somewhat effective  ⬜ 2 | Effective  ⬜ 3 | Very effective  ⬜ 4 |

Please include any other comments:

Adapted from a similar survey from Kelley AS, Back AL, Arnold RM, et al. Geritalk: communication skills training for geriatric and palliative medicine fellows. J Am Geriatr Soc. 2012;60(2):332-337. doi:10.1111/j.1532-5415.2011.03787.x
