# Supplementary material for: Eating Problems in Advanced Dementia: Navigating Difficult Conversations
Source: MedEdPORTAL. 2020 Nov 17;16:11025. doi: 10.15766/mep_2374-8265.11025 (PMC7678029; doi:10.15766/mep_2374-8265.11025)
Supplement: Supplementary file 1 — Facilitator Guide.docxParticipant Completed Worksheet.docxParticipant Handout.docxVideo.mp4Learning Objectives and Case.docxParticipant Blank Worksheet.docxParticipant Survey.docx [file mep_2374-8265.11025-s001.zip › _Educational_Summary_Report_11025.pdf]

MedEdPORTAL® The AAMC Journal of Teaching and Learning Resources

Original Publication Open Access

Eating Problems in Advanced Dementia: Navigating Difficult Conversations

Erika R. Manu, MD*, James T. Fitzgerald, PhD, Patricia B. Mullan, PhD, Caroline A. Vitale, MD

*Corresponding author: erikaman@med.umich.edu

Abstract

Introduction: The majority of older adults with advanced dementia (AD) develop difficulties with eating and swallowing, often prompting concerns about nutrition and quality of life. Employing a palliative approach requires providers to attain skills in addressing symptoms and communicating with family caregivers about the trajectory of AD and associated dysphagia, as well as to elicit goals of care. Research suggests internal medicine (IM) residents often perceive minimal education during training addressing skills needed to care for patients with AD. **Methods:** We developed and piloted a small-group interactive seminar utilizing a trigger video depicting a family meeting addressing eating problems in a patient with AD. Case-based learning, small-group discussion, and learner reflection were employed. We assessed the impact on 82 of the 106 IM, medicine-pediatrics, and neurology residents who participated in the seminar. **Results:** Participant evaluation indicated residents showed high satisfaction and perceived the educational content of the seminar to be robust and clinically relevant. We found statistically significant ($p < .001$) improvements in self-reported confidence in dementia-specific skills postseminar. Effect size was large to very large (Cohen's $d = 1.3$-1.7). **Discussion:** An interactive, case-based seminar utilizing a video depicting a realistic family meeting improved residents' self-efficacy in skills needed to address nutritional issues, engage in goals-of-care discussions, and reflect on concerns among caregivers of patients with AD. The seminar teaches important geriatric and palliative concepts meant to improve residents' ability to care for older adults with AD in their future careers.

Keywords
Resident Education, Palliative Care Education, Geriatrics Education, Advanced Dementia, Dysphagia, Geriatrics, Hospice & Palliative Medicine, Case-Based Learning

Educational Objectives

By the end of this session, learners will be able to:

1. Identify the nature and causes of eating problems in a patient with advanced dementia.
2. Describe potential treatment burdens associated with artificial nutrition in patients with advanced dementia.
3. Discuss various options for addressing eating/feeding difficulties in patients with advanced dementia.
4. Identify and address challenging beliefs and concerns posed by the family/caregivers.

Introduction

Advanced dementia (AD) is increasingly recognized as a terminal illness associated with substantial symptom burden and care needs.[1] Competent care of patients with AD requires the ability to recognize the clinical manifestations of AD and the skills to address the changing care needs that arise among patients in the advanced stage. Persistent eating problems are considered a hallmark of AD[1] and can prompt serious concerns among family caregivers and medical providers about nutrition, potential risk of aspiration, and patient preferences, along with considerations of comfort and quality of life. Our prior research suggests that internal medicine (IM) residents often feel they lack key skills important for optimal care of patients with AD.[2] While IM residents view these skills as important for their future careers, they report feeling somewhat unprepared to manage this patient population. This work has delineated the need to develop further geriatric and palliative care curricula during residency training focused on how to best provide high-quality, goal-concordant care for older adults with neurodegenerative conditions such as dementia and their caregivers.

As of January 2016, the Center for Medicare and Medicaid Services pays for voluntary advance care planning (ACP) in recognition of its importance for providing care aligned with

Citation:
Manu ER, Fitzgerald JT, Mullan PB, Vitale CA. Eating problems in advanced dementia: navigating difficult conversations. *MedEdPORTAL*. 2020;16:11025.
https://doi.org/10.15766/mep_2374-8265.11025

Copyright © 2020 Manu et al. This is an open-access publication distributed under the terms of the Creative Commons Attribution license.

patients' goals and preferences, including care at the end of life.[3] For patients diagnosed with dementia, ACP should include an early conversation about the possibility of developing eating problems in the advanced stages of the disease, along with an exploration of patient preferences and values should eating problems arise. In support of this very important topic, the American Geriatrics Society, in response to the Choosing Wisely campaign, included a recommendation to avoid feeding tube insertion in patients with AD and, instead, offer careful hand-feeding in its list of 10 items meant to promote patient-physician conversations.[4] This initiative has provided added impetus to our work.

The purpose of our work was to develop and implement an interactive small-group seminar utilizing a trigger video depicting a family meeting addressing eating problems that have arisen in a patient with AD. The seminar aim is to elucidate the importance of employing a thoughtful, nuanced, and evidenced-based approach to the multifaceted clinical dilemma of persistent and progressive eating problems in AD. We sought to assess the impact of this seminar on IM, medicine-pediatrics, and neurology residents' self-perceived confidence in addressing these concerns with surrogate decision-makers of patients with AD, as well as to elicit the residents' perception of the educational value of the seminar.

Our work aligns with the Institute for Healthcare Improvement and the John A. Hartford Foundation's effort to promote age-friendly heath systems utilizing the 4M framework, in which particular attention should be given to the following: carefully eliciting what Matters to older adults, appropriate Medication use, assessing cognition (Mentation), and Mobility, in order to provide high-quality geriatric care.[5] This seminar especially addresses the Mentation and what Matters aspects of the 4Ms framework, enhancing learners' awareness of the importance of assessing these areas when caring for older adults in any setting.

Other *MedEdPORTAL* resources that address some concepts overlapping those covered by this small-group seminar are available. One *MedEdPORTAL* publication covers the larger concept of ACP, which includes ACP for older adults with dementia.[6] Another considers dementia as a cause of weight loss among older adults.[7] They both employ role-playing and simulation as instructional methods, requiring prework for participants and facilitators, as well as a simulation lab and standardized patients. In contrast to these resources, our seminar is dedicated in its entirety to discussing the development of eating and swallowing problems in the advanced stage of dementia, along with concerns among family caregivers, and requires minimal financial or structural resources or learner prework for implementation.

Methods

Seminar Development

We developed the original materials and recorded a trigger video simulating a family meeting with actors (including one professional actor and two amateur actors) playing the roles of a physician, a speech-language pathologist, and the daughters of a patient with AD. The video modeled an approach for assessing and addressing eating problems in AD. It was 14 minutes long and contained questions intended to trigger participants' input. We developed a facilitator guide (Appendix A) to train faculty clinician educators to lead the seminar along with two participant handouts (Appendices B and C) featuring topic-related content. Within the facilitator guide, there were several evidence-based principles upon which the facilitator could draw in preparation for guiding the small-group discussion.[8-27] Funding for this project was provided in part by a grant from the Center for Research on Learning and Teaching (CRLT) at the University of Michigan and by the John A. Hartford Foundation.

Description of the Seminar

Our monthly seminar was held during a recurring time slot within the IM/medicine-pediatrics academic Thursday afternoon didactic sessions. The location was usually a medical school classroom, equipped with a computer and a video projector. The video (Appendix D) was the centerpiece of this interactive small-group seminar lasting 90 minutes. We started the seminar with a review of the learning goals, followed by an introduction of the topic utilizing a written case (Appendix E) read by the faculty facilitator or a participant. We then played the video, stopping it for discussion each time a trigger question was encountered. Utilizing these trigger questions to open the conversation and stimulate discussion among the participants, we asked learners to contemplate clinical and ethical issues elucidated by the video; to share their knowledge, thoughts, and perceptions; and to ask questions. We provided participants with a blank worksheet (Appendix F) to complete while taking part in the seminar, which they kept at the end of the seminar. We encouraged learners to exchange ideas through active discussion while we addressed knowledge gaps and questions by facilitating the discussion. At the end of the seminar, we provided participants with two handouts (Appendices B and C) synthesizing the information discussed and asked them to complete a short postseminar survey (Appendix G).

Copyright © 2020 Manu et al. This is an open-access publication distributed under the terms of the Creative Commons Attribution license.

Target Learners

We implemented the seminar during a mandatory monthly geriatric seminar series imbedded within the IM/medicine-pediatrics curriculum, with 10-12 second-, third-, and fourth-year residents for a 6-month period. However, based on participant feedback, we subsequently introduced the seminar earlier in the residents' training, targeting first-year IM and medicine-pediatrics residents, with four interns participating each month for the next 2 years and 3 months. Ten to 12 neurology residents also attended the seminar during their July boot camp. The Institutional Review Board at the University of Michigan approved our project as exempt (HUM 00091437, approved as exempt: September 1, 2016).

Evaluation

We developed a retrospective pre/post survey (Appendix G) to assess perceived skills in five areas along with the perceived educational value of the seminar. The survey was based on a previously published one.[28] Residents rated their abilities to discuss eating problems in AD using a Likert scale that indicated their perceived level of independence (1 = *need further instruction,* 2 = *with supervision,* 3 = *with backup,* 4 = *unsupervised*). We also assessed the perceived educational impact of the seminar utilizing a 4-point rating scale. At the end of the survey, we provided the option for free-text feedback. The survey was not linked to a course grade.

Data Analysis

We utilized paired *t* tests (two-tailed) to examine change in confidence levels. Cohen's *d* was used to calculate effect sizes.

Results

A total of 106 residents participated in the seminar, with 82 completing the postseminar assessment. Our response rate was 77% (82 of 106). A statistically significant difference ($p < .001$) was found in the pre/post self-reported confidence scores in all five skills (Figure 1). Cohen's *d* indicated large effect sizes (Table). The seminar educational value ratings are depicted in Figure 2. Residents rated the seminar favorably, 3.6-3.7 on a 4-point scale.

Open-ended text feedback from participant learners also included the following comments grouped along the following themes:

- Clinical relevance:
  - "This is a very recurrent topic, important to formally go over."
  - "This workshop should have happened at the beginning of my residency."
  - "This would likely be helpful for the interns."
  - "Very helpful, has come up previously, I did not know how to really handle it."
  - "Really important topic, we see this all the time and often don't know the data."
- Group dynamics:

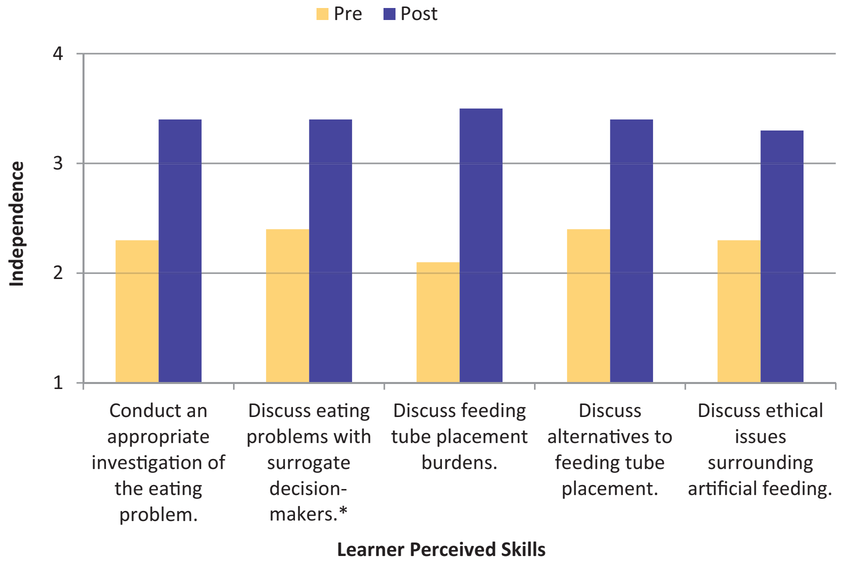

Figure 1. Learner perceived confidence with five skills needed to care for patients with advanced dementia before and after seminar completion ($N = 82$; * indicates $N = 81$). For all comparisons, $p < .001$. Rated on an independence scale (1 = *need further instruction,* 2 = *with supervision,* 3 = *with backup,* 4 = *unsupervised*).

Copyright © 2020 Manu et al. This is an open-access publication distributed under the terms of the Creative Commons Attribution license.

MedEdPORTAL® The AAMC Journal of Teaching and Learning Resources

Table. Pre- and Postseminar Score Differences (Paired *t* Test)

Skill Confidence Question[a]	Pre/Post Mean Difference	Difference *SD*	95% CI	Effect Size[b]
Conduct an appropriate investigation of the eating problem.	−1.09	.69	−1.2 to −0.9	1.6
Discuss eating problems with surrogate decision-makers.[c]	−1.01	.70	−1.1 to −0.8	1.4
Discuss feeding tube placement burdens.	−1.39	.84	−1.5 to −1.2	1.7
Discuss alternatives to feeding tube placement.	−1.09	.82	−1.2 to −0.9	1.3
Discuss ethical issues surrounding artificial feeding.	−1.05	.83	−1.2 to −0.8	1.3

[a] N = 82, except where indicated.
[b] Cohen's *d*: small effect = 0.2, medium effect = 0.5, and large effect= 0.8.
[c] N = 81.

  - "Group discussion and interactions was key. Anything to facilitate more of this is great."
  - "Liked that we were able to discuss things further than only the provided questions."
- Seminar format/quality of video:
  - "I liked how realistic the video was, as well as the response and question format."
  - "Excellent workshop, valuable teaching points presented in a multimodal format which helped cement the information."
  - "The video was very useful in guiding discussions."
  - "Good interaction with the video."

Discussion

This interactive seminar utilizing an educational trigger video and small-group discussion improved IM residents' self-perceived confidence in five skills needed to care for patients with AD and eating and swallowing problems. The seminar showed a substantial effect on improving residents' confidence in performing these skills and was well received. In addition, based on qualitative feedback from learners suggesting the seminar would have been helpful to have earlier on during training, we were subsequently successful in adjusting the timing of the seminar to occur during intern year.

The intervention requires the faculty facilitator to review the facilitator guide but does not require other in-depth preparation, making it feasible to implement within various educational settings and/or curricula. The embedded trigger questions promote participants' sharing of knowledge and viewpoints through guided discussion; group discussion is followed by distribution of an evidence-based, referenced summary of content covered during the session. The seminar discussion is enhanced by the faculty facilitator sharing clinical, experiential, and evidence-based knowledge (the latter based on content covered in the facilitator guide) where needed.

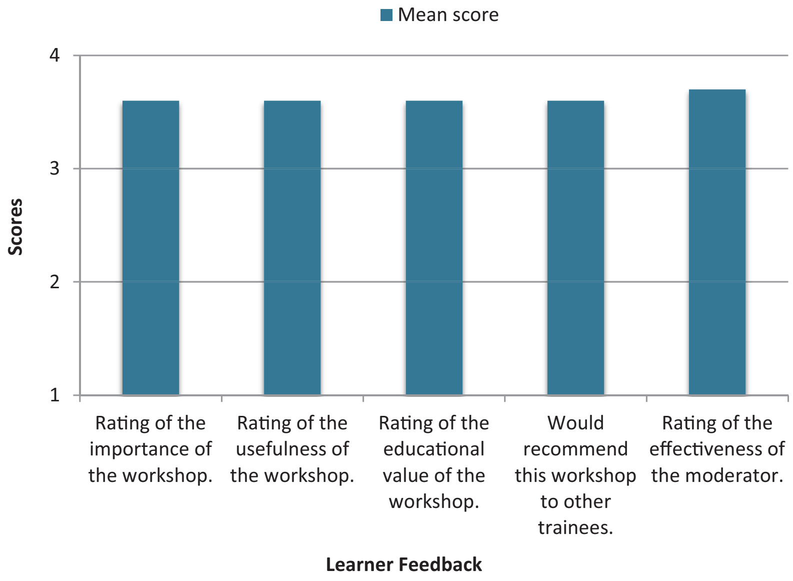

Figure 2. Leaner perceived educational value of the seminar (N = 82).

Copyright © 2020 Manu et al. This is an open-access publication distributed under the terms of the Creative Commons Attribution license.

The video depicts a realistic family meeting that includes a speech pathologist, a physician, and two adult daughters of the patient, each with separate concerns and perspectives. This simulated family meeting serves as a focal point for discussion and illustrates a realistic goals-of-care conversation. The goal is to have the learners discuss various approaches and strategies that can be utilized when confronted with family members displaying a host of emotions, including sadness, anger, and distrust, as well as gratitude and appreciation. We believe this interactive seminar provides a venue in which learners can consider different perspectives and gain insight into areas of possible distress that caregivers often experience when attempting to make the most informed decisions possible on behalf of their loved ones. The trigger video depicts caregivers who seek to gain a clear understanding of their loved one's medical condition while modeling providers who are utilizing evidence-based approaches to assessing eating problems in patients with AD.

A study by Hanson and colleagues[29] showed that using a decision aid for goals-of-care conversations was helpful in empowering surrogate decision-makers to make more informed decisions for their loved ones with AD. Nonetheless, physician and interprofessional team communication may still account for gaps in quality care at the end of life in patients with AD.[30] We believe this interactive seminar helps to empower providers to address caregiver concerns by offering important insight into caregivers' experiences, perspectives, and emotions that often color the surrogate decision-making process. Allowing learners to deconstruct and discuss discrete aspects of the family meeting depicted in the video while in a safe small-group learning environment is helpful especially when asking them to consider scenarios using alternate language and phrasing and teaching them to look for and anticipate emotional cues. This small-group seminar helps prepare providers to successfully lead nuanced goals-of-care discussions and ultimately to improve care for patients with AD and eating difficulties.

Strengths and Limitations

Strengths: This seminar addresses an identified gap in resident education by providing the opportunity to discuss the difficult topic of eating and swallowing problems commonly occurring in patients with AD in an interactive small-group setting. The facilitator has to complete prework, including reading the facilitator guide and printing out the handouts/materials, but the participants are not required to do any prework. The length of the session facilitates its integration into an existing resident curriculum as a recurring small-group seminar, perhaps within a dedicated didactic afternoon block, as we did at our program. The seminar can also be easily delivered to other interprofessional learners; we have been successful in delivering it without any modification to social work students, as an example. The seminar does not require special technology beyond audiovisual computer technology (PC and a projector) and a few printed materials/handouts.

Limitations/lessons learned: We note that the approach exemplified in the video is meant to stimulate discussion with learners and is not necessarily a depiction of an ideal family meeting or goals-of-care discussion. However, we believe it illustrates a realistic family meeting and provides plenty of material for discussion covering evidence-based content and consideration of important qualitative and emotional aspects of these discussions.

We did not stratify our learners by specialty or year of training, but we received feedback from the residents suggesting earlier implementation would be helpful; therefore, we were able to change the timing of our seminar to include first-year residents, who provided the majority of the responses. Another limitation is that we did not incorporate a knowledge check as part of the assessment, both in an effort to limit the length of the assessment and due to the lack of existing validated questions that we felt would appropriately assess participants' knowledge gained. We also found that smaller groups of learners (four to five participants) were better suited for this seminar than the larger groups of 10-12, to allow for more inclusive and active participation and less anxiety with sharing personal experiences.

While we found the free-text comments valuable and insightful, the overall low number of them did not allow for further identification of themes beyond the three categories listed above.

Additionally, we are not able to report patient-level outcomes or point to reliable evidence of practice change because we believe these outcomes are usually influenced by a multitude of factors related to the residents' clinical training, so that attributing a change to residents' attendance at our seminar would be difficult. Subsequent follow-up with participants was cost-prohibitive due to limited resources. However, we do report robust effect sizes gleaned from participants' retrospective pre/post seminar data encompassing important skill domains integral to delivering high-quality care to persons with dementia.

One primary facilitator with geriatric expertise provided most of the sessions. Therefore, we are unable to fully comment on faculty facilitator perceptions of the ease of facilitating

Copyright © 2020 Manu et al. This is an open-access publication distributed under the terms of the Creative Commons Attribution license.

the seminar. We do, however, recommend that facilitators become familiar with the material provided in the guide before implementing the seminar.

Our initial intention with the participant blank worksheet (Appendix F) was to allow participants to compare their responses with the completed version (Appendix B), but experience showed low utilization of the blank worksheet by learners. Therefore, although we feel facilitators could consider forgoing the blank worksheet's use, some learners may appreciate being prompted by it in order to provide a framework for the anticipated flow of the video and discussion. Lastly, we suggest facilitators have the video downloaded and ready to play for the seminar, to minimize interruptions.

Future Directions
As we have now ended these seminar sessions due to curricular changes in the IM residency program, we intend to adapt the seminar content to include interprofessional competencies (e.g., teamwork, communications skills, and roles and responsibilities). The topic of feeding problems in AD remains of interest to many health care providers, and we plan to target nursing, nurse practitioner, and social work learners, among others, within our medical centers.

In closing, we feel this seminar is a useful tool to model ways to address a difficult, emotionally laden topic that continues to be relevant given our aging population. The seminar offers an educational venue to help prepare clinicians to provide high-quality care for older adults within age-friendly heath systems.

Appendices

A. Facilitator Guide.docx
B. Participant Completed Worksheet.docx
C. Participant Handout.docx
D. Video.mp4
E. Learning Objectives and Case.docx
F. Participant Blank Worksheet.docx
G. Participant Survey.docx

All appendices are peer reviewed as integral parts of the Original Publication.

Erika R. Manu, MD: Assistant Professor, Division of Geriatric and Palliative Medicine, Department of Internal Medicine, University of Michigan Medical School; Physician, VA Ann Arbor Geriatric Research, Education and Clinical Center (GRECC)

James T. Fitzgerald, PhD: Professor Emeritus, Department of Learning Health Sciences, University of Michigan Medical School

Patricia B. Mullan, PhD: Professor Emerita, Department of Learning Health Sciences, University of Michigan Medical School

Caroline A. Vitale, MD: Professor, Division of Geriatric and Palliative Medicine, Department of Internal Medicine, University of Michigan Medical School; Associate Director of Education and Evaluation, VA Ann Arbor Geriatric Research, Education and Clinical Center (GRECC)

Acknowledgments
Dr. Kenneth Pituch provided important support for developing the idea of this project based on a similar pediatric workshop design he had previously developed and implemented.

Disclosures
None to report.

Funding/Support
Funded in part by the University of Michigan Center for Research on Learning and Teaching through the Faculty Development Fund (2014) and the John A. Hartford Foundation.

Prior Presentations
Manu E, Vitale C, Murray J, Lustig A. When eating problems arise in patients with advanced dementia—an interdisciplinary approach to communicating with caregivers. Workshop presented at: American Association of Hospice and Palliative Medicine Annual Meeting; February 25-28, 2015; Philadelphia, PA.

Manu E, Dengiz A, Fitzgerald T, Vitale C. When eating problems arise in patients with advanced dementia—an interdisciplinary approach to communicating with caregivers. Workshop presented at: American Geriatrics Society (AGS) 2016 Annual Scientific Meeting; May 19-21, 2016; Long Beach, CA.

Ethical Approval
The University of Michigan Institutional Review Board approved this study.

Disclaimer
The views expressed in this publication are the authors' own opinions and not an official position of the Veterans Affairs or of the University of Michigan Medical School.

References

1. Mitchell SL, Teno JM, Kiely DK, et al. The clinical course of advanced dementia. *N Engl J Med*. 2009;361(16):1529-1538. https://doi.org/10.1056/NEJMoa0902234
2. Manu E, Marks A, Berkman CS, Mullan P, Montagnini M, Vitale CA. Self-perceived competence among medical residents in skills needed to care for patients with advanced dementia versus

Copyright © 2020 Manu et al. This is an open-access publication distributed under the terms of the Creative Commons Attribution license.

metastatic cancer. *J Cancer Educ*. 2012;27(3):515-520. https://doi.org/10.1007/s13187-012-0351-2

3. MLN fact sheet: advance care planning. *Centers for Medicare and Medicaid Services*. August 2019. https://www.cms.gov/Outreach-and-Education/Medicare-Learning-Network-MLN/MLNProducts/Downloads/AdvanceCarePlanning.pdf
4. Feeding tubes for people with Alzheimer's disease: when you need them—and when you don't. *Choosing Wisely*. 2017. https://www.choosingwisely.org/wp-content/uploads/2018/02/Feeding-Tubes-For-People-With-Alzheimers-AGS.pdf
5. Age-friendly health systems initiative. John A. Hartford Foundation. Accessed October 2, 2020. https://www.johnahartford.org/grants-strategy/current-strategies/age-friendly/age-friendly-health-systems-initiative
6. Grey C, Constantine L, Baugh GM, Lindenberger E. Advance care planning and shared decision-making: an interprofessional role-playing workshop for medical and nursing students. *MedEdPORTAL*. 2017;13:10644. https://www.mededportal.org/doi/10.15766/mep_2374-8265.10644
7. Mulligan R, Gilmer-Scott M, Kouchel D, et al. Unintentional weight loss in older adults: a geriatric interprofessional simulation case series for health care providers. *MedEdPORTAL*. 2017;13:10631. https://www.mededportal.org/doi/10.15766/mep_2374-8265.10631
8. Smith AK, Lo B, Sudore R. When previously expressed wishes conflict with best interests. *JAMA Intern Med*. 2013;173(13): 1241-1245. https://doi.org/10.1001/jamainternmed.2013.6053
9. Vitale CA, Monteleoni C, Burke L, Frazier-Rios D, Volicer L. Strategies for improving care for patients with advanced dementia and eating problems. *Ann Longterm Care*. 2009;17(5):32-39.
10. Ney DM, Weiss JM, Kind AJH, Robbins J. Senescent swallowing: impact, strategies, and interventions. *Nutr Clin Pract*. 2009; 24(3):395-413. https://doi.org/10.1177/0884533609332005
11. Meier DE, Ahronheim JC, Morris J, Baskin-Lyons S, Morrison RS. High short-term mortality in hospitalized patients with advanced dementia: lack of benefit of tube feeding. *Arch Intern Med*. 2001; 161(4):594-599. https://doi.org/10.1001/archinte.161.4.594
12. Teno JM, Gozalo P, Mitchell SL, Kuo S, Fulton AT, Mor V. Feeding tubes and the prevention or healing of pressure ulcers. *Arch Intern Med*. 2012;172(9):697-701. https://doi.org/10.1001/archinternmed.2012.1200
13. Kuo S, Rhodes RL, Mitchell SL, Mor V, Teno JM. Natural history of feeding-tube use in nursing home residents with advanced dementia. *J Am Med Dir Assoc*. 2009;10(4):264-270. https://doi.org/10.1016/j.jamda.2008.10.010
14. Ijaopo EO, Ijaopo RO. Tube feeding in individuals with advanced dementia: a review of its burdens and perceived benefits. *J Aging Res*. 2019;2019:7272067. https://doi.org/10.1155/2019/7272067
15. Takayama K, Hirayama K, Hirao A, et al. Survival times with and without tube feeding in patients with dementia or psychiatric diseases in Japan. *Psychogeriatrics*. 2017;17(6):453-459. https://doi.org/10.1111/psyg.12274
16. Finucane TE, Christmas C, Travis K. Tube feeding in patients with advanced dementia: a review of the evidence. *JAMA*. 1999; 282(14):1365-1370. https://doi.org/10.1001/jama.282.14.1365
17. Langmore SE, Skarupski KA, Park PS, Fries BE. Predictors of aspiration pneumonia in nursing home residents. *Dysphagia*. 2002;17(4):298-307. https://doi.org/10.1007/s00455-002-0072-5
18. Teno JM, Gozalo PL, Bynum JPW, et al. Change in end-of-life care for Medicare beneficiaries: site of death, place of care, and health care transitions in 2000, 2005, and 2009. *JAMA*. 2013; 309(5):470-477. https://doi.org/10.1001/jama.2012.207624
19. Givens JL, Selby K, Goldfeld KS, Mitchell SL. Hospital transfers of nursing home residents with advanced dementia. *J Am Geriatr Soc*. 2012;60(5):905-909. https://doi.org/10.1111/j.1532-5415.2012.03919.x
20. Carey TS, Hanson L, Garrett JM, et al. Expectations and outcomes of gastric feeding tubes. *Am J Med*. 2006;119(6): 527.e11-527.e16. https://doi.org/10.1016/j.amjmed.2005.11.021
21. Sanders DS, Carter MJ, D'Silva J, James G, Bolton RP, Bardhan KD. Survival analysis in percutaneous endoscopic gastrostomy feeding: a worse outcome in patients with dementia. *Am J Gastroenterol*. 2000;95(6):1472-1475.
22. Goldberg LS, Altman KW. The role of gastrostomy tube placement in advanced dementia with dysphagia: a critical review. *Clin Interv Aging*. 2014;9:1733-1739. https://doi.org/10.2147/CIA.S53153
23. Hanson LC, Ersek M, Gilliam R, Carey TS. Oral feeding options for people with dementia: a systematic review. *J Am Geriatr Soc*. 2011;59(3):463-472. https://doi.org/10.1111/j.1532-5415.2011.03320.x
24. McCann RM, Hall WJ, Groth-Juncker A. Comfort care for terminally ill patients: the appropriate use of nutrition and hydration. *JAMA*. 1994;272(16):1263-1266. https://doi.org/10.1001/jama.1994.03520160047041
25. Snyder EA, Caprio AJ, Wessell K, Lin FC, Hanson LC. Impact of a decision aid on surrogate decision-makers' perceptions of feeding options for patients with dementia. *J Am Med Dir Assoc*. 2013;14(2):114-118. https://doi.org/10.1016/j.jamda.2012.10.011
26. Mitchell SL, Shaffer ML, Cohen S, Hanson LC, Habtemariam D, Volandes AE. An advance care planning video decision support tool for nursing home residents with advanced dementia: a cluster randomized clinical trial. *JAMA Intern Med*. 2018;178(7):961-969. https://doi.org/10.1001/jamainternmed.2018.1506
27. Palecek EJ, Teno JM, Casarett DJ, Hanson LC, Rhodes RL, Mitchell SL. Comfort feeding only: a proposal to bring clarity to

Copyright © 2020 Manu et al. This is an open-access publication distributed under the terms of the Creative Commons Attribution license.

decision-making regarding difficulty with eating for persons with advanced dementia. *J Am Geriatr Soc*. 2010;58(3):580-584. https://doi.org/10.1111/j.1532-5415.2010.02740.x

28. Kelley AS, Back AL, Arnold RM, et al. Geritalk: communication skills training for geriatric and palliative medicine fellows. *J Am Geriatr Soc*. 2012;60(2):332-337. https://doi.org/10.1111/j.1532-5415.2011.03787.x

29. Hanson LC, Zimmerman S, Song MK, et al. Effect of the goals of care intervention for advanced dementia: a randomized clinical trial. *JAMA Intern Med*. 2017;177(1):24-31. https://doi.org/10.1001/jamainternmed.2016.7031

30. Biola H, Sloane PD, Williams CS, Daaleman TP, Williams SW, Zimmerman S. Physician communication with family caregivers of long-term care residents at the end of life. *J Am Geriatr Soc*. 2007;55(6):846-856. https://doi.org/10.1111/j.1532-5415.2007.01179.x

Received: December 17, 2019
Accepted: July 2, 2020
Published: November 17, 2020

Copyright © 2020 Manu et al. This is an open-access publication distributed under the terms of the Creative Commons Attribution license.
